# Primary Metabolites in Three *Ocimum* Species: Compositional Diversity, Network Pharmacology, and Integrin-Targeted Therapeutic Implications

**DOI:** 10.3390/life16020273

**Published:** 2026-02-04

**Authors:** Jingtian Yang, Jialin Li, Mei Liu, Yanping Mao, Ruijun Su, Cong Zhao, Jian Yang, Qinggui Wu, Yi Huang

**Affiliations:** 1 Forest Ecology and Conservation in the Upper Reaches of the Yangtze River Key Laboratory of Sichuan Province, Engineering Research Center for Forest and Grassland Disaster Prevention and Reduction at Mianyang Normal University of Sichuan Province, School of Life Sciences, College of Biology and Pharmacy, Mianyang Normal University, Mianyang 621000, China; 2 School of Environmental Science and Engineering, Southwest Jiaotong University, Chengdu 611756, China; 3 Sichuan Provincial Forest and Grassland Key Laboratory of Alpine Grassland Conservation and Utilization of Tibetan Plateau, College of Grassland Resources, Southwest Minzu University, Chengdu 610093, China; 4 China College of Science, Tibet University, Lhasa 850012, China

**Keywords:** *Ocimum*, primary metabolites, multi-omics, integrin signaling, network pharmacology, UPLC-MS/MS

## Abstract

*Ocimum* (basil) is a globally significant medicinal and culinary herb. While its bioactive secondary metabolites are well-studied, the medicinal potential of its abundant primary metabolites (amino acids, vitamins, carbohydrates, steroids) remains largely unexplored. To address this gap, we employed an integrated multi-omics strategy. First, UPLC-MS/MS-based metabolomics quantified primary metabolites across six distinct *Ocimum* accessions (*Ocimum* × africanum, *Ocimum tenuiflorum*, *Ocimum gratissimum*). Profiling identified 291 primary metabolites, revealing significant interspecific variation, with 273 differential accumulated metabolites (DAMs). Subsequent network pharmacology analysis of 61 high-impact DAMs predicted 516 potential targets. Protein–protein interaction refinement yielded 28 core targets, predominantly integrins (ITGB1, ITGB3, ITGA4, ITGA2B, ITGAV) and kinases (IGF1R, PIK3CA, SRC). Enrichment analysis implicated these targets in focal adhesion, ECM-receptor interaction, and PI3K-Akt signaling pathways. Molecular docking confirmed strong potential binding (binding energy < −7 kcal/mol) between key tripeptides (e.g., Met-Ser-Tyr, Phe-Cys-Gln) and integrin subunits. Antioxidant assays (DPPH, ABTS, FRAP) further showed significant genotypic variation. This study systematically deciphers the primary metabolome of *Ocimum* and, through a multi-omics approach, reveals novel integrin-mediated mechanisms underpinning its potential therapeutic value, providing a foundation for developing basil-based nutraceuticals and pharmaceuticals.

## 1. Introduction

*Ocimum,* commonly known as basil, is an annual or perennial herbaceous plant belonging to the Lamiaceae family. Originating from tropical regions of Africa and Asia, it is now cultivated globally in warm climates, including numerous provinces in southern China [1]. Characterized by erect stems, opposite leaves, and a distinctive pungent aroma derived from volatile terpenoids, basil holds substantial economic value as a versatile plant used in culinary, medicinal, and industrial applications [2,3]. Its role in traditional medical systems—notably Ayurveda and Traditional Chinese Medicine (TCM)—is well-documented, with reported properties including dispelling wind-cold, invigorating the spleen to resolve dampness, and detoxifying to reduce swelling [4,5]. Modern scientific research has validated these traditional uses and further uncovered a spectrum of bioactivities, such as potent antioxidant, anti-inflammatory, antimicrobial, and neuroprotective effects [6].

As an important aromatic and traditional medicinal plant, basil’s significant medicinal value is widely recognized [7]. To date, research has primarily focused on plant secondary metabolites—such as phenolics, terpenes, and flavonoids—as the core phytochemical basis underlying its diverse bioactivities [8,9]. Consequently, extensive efforts have been devoted to isolating, identifying, and elucidating the pharmacological mechanisms of these secondary metabolites, greatly advancing our understanding of basil’s therapeutic potential [10].

However, amid this focus on secondary metabolism, the intrinsic value of basil’s equally abundant and essential primary metabolites has been relatively overlooked [11]. These fundamental compounds, including amino acids (the building blocks of life) [12], vitamins (crucial for key physiological processes) [13], carbohydrates (providing energy and structural support) [14], and steroid compounds (precursors to important bioactive molecules) [15] are often relegated to the status of mere background constituents essential for growth and development, without sufficient evaluation of their direct contributions or synergistic roles within the plant’s overall medicinal efficacy profile [16].

In reality, primary metabolites are not only fundamental to basic plant physiology but also possess significant inherent physiological regulatory functions [17]. This bioactivity is evident across various metabolite classes present in basil. For instance, free amino acids and peptides contribute substantially to its medicinal profile. Basil leaves contain considerable levels of free amino acids, with specific constituents like γ-aminobutyric acid demonstrating documented hypotensive and cognitive-enhancing effects [18,19]. Notably, bioactive oligopeptides, such as the tripeptide Ile-His-Val, exhibit significant antioxidant and anti-inflammatory activities, including free radical scavenging and inhibition of pro-inflammatory cytokine release. The potent ACE inhibitory activity of Ile-His-Val underscores the potential of such small, bioavailable peptides as valuable leads for novel therapeutics [20,21,22].

The vitamin composition of basil further underscores the functional role of primary metabolites. Basil is rich in both lipophilic vitamins, such as Vitamin E, and hydrophilic vitamins like Vitamin C [23,24]. These vitamins act synergistically within antioxidant systems (e.g., the redox interplay between Vitamins C and E) and serve as essential cofactors in enzymatic reactions (as seen with B vitamins), collectively providing critical cellular protection against oxidative damage [25].

Steroid compounds, including phytosterols like β-sitosterol and stigmasterol, are also present and are known for their cholesterol-lowering potential partly mediated through mechanisms such as HMG-CoA reductase inhibition and anti-tumor activity [26]. Beyond these roles, steroid components may exert anti-inflammatory effects by modulating pathways like the hypothalamic–pituitary–adrenal (HPA) axis or via glucocorticoid-mimetic actions. Additionally, basil saponins contribute to antitumor effects, as evidenced by activity against HeLa cells [27,28].

Finally, complex polysaccharides in basil, primarily composed of arabinose (42%), galactose (31%), and rhamnose (15%) with a molecular weight range of 5–30 kDa, exhibit immunomodulatory and potential prebiotic effects [29]. This is supported by their ability to stimulate immune responses, such as promoting nitric oxide (NO) production in macrophages, suggesting significant roles in gut health and systemic immune regulation [19,30].

Furthermore, emerging evidence supports a network pharmacology paradigm. Studies, such as those demonstrating that basil flower extract ameliorates lung injury through modulation of the NLRP3 inflammasome signaling pathway [31], underscore that bioactivity often arises from complex interactions of multiple components acting on diverse targets. This reinforces the need for a holistic view of plant metabolites, where primary metabolites likely participate in these intricate networks [32,33]. The systematic neglect of these foundational primary metabolites inevitably restricts a comprehensive understanding of the mechanisms underpinning basil’s medicinal value and risks overlooking potential novel applications based on these compounds.

Therefore, systematic investigation into the composition, quantification, and bioactivity evaluation of primary metabolites amino acids, vitamins, carbohydrates, and steroids in basil is imperative [32]. This research serves not merely as a supplement to the existing secondary metabolite-centric studies but is fundamental to holistically deciphering the totality of basil’s medicinal value, unearthing novel health-promoting functions, and providing a robust scientific foundation for its comprehensive development and utilization. This study directly addresses this critical knowledge gap [34,35].

Leveraging basil’s status as a medicinal and edible plant and the complexity of its metabolic network, this study employs an innovative multi-omics approach. UPLC-MS/MS-based metabolomics: For precise, panoramic quantification of primary metabolites (amino acids, vitamins, carbohydrates, sterols) across six distinct basil genotypes. Network pharmacology was used to systematically analyze the medicinal value and potential mechanisms of action of candidate differential metabolites, thereby identifying key targets and pathways [36]. Molecular docking was performed to elucidate the specific binding interactions and affinities between critical differential metabolites and core target proteins, providing mechanistic insights at the atomic level [37]. Integrated metabolite-target-disease network analysis: Synthesizing data from the above approaches to construct a multi-dimensional regulatory network, offering novel perspectives for functional ingredient development.

This integrated strategy aims to comprehensively elucidate the profile of primary metabolites in basil and their associated medicinal potential. Consequently, our findings will establish a robust scientific foundation enabling: (1) the expansion of basil’s applications in pharmaceutical, nutraceutical, and cosmetic sectors; (2) the provision of a novel paradigm for the development of medicinal-and-edible plants; (3) significant contributions of innovative case studies and methodologies to plant metabolomics and network pharmacology; and (4) the facilitation of innovative drug target discovery from primary metabolic pathways. Ultimately, this research seeks to deepen our understanding of this valuable medicinal resource while propelling advancements in related scientific disciplines.

## 2. Materials and Methods

### 2.1. Plant Materials

The experimental materials comprised six accessions from three *Ocimum* species: *Ocimum* × africanum (G061, G072), *Ocimum* tenuiflorum (G060, G085), and *Ocimum* gratissimum (G134, G144), with two accessions per species. All plants were cultivated under standardized conditions at the Nanyao Germplasm Resources Nursery, Institute of Tropical Crop Variety Resources, Chinese Academy of Tropical Agricultural Sciences, Danzhou, Hainan Province, China. For each accession, nine healthy, uniform, and disease-free plants were systematically selected as experimental subjects to ensure statistical reliability. These plants were grouped into three biological replicates (three plants per replicate). Mature leaves from 180-day-old each plant were promptly flash-frozen in liquid nitrogen and stored at −80 °C until subsequent analysis.

### 2.2. Metabolite Identification

#### 2.2.1. Sample Preparation

The biological samples underwent vacuum freeze-drying for 63 h in a Scientz-100F freeze dryer (Ningbo Scientz Biotechnology Co., Ltd., Ningbo, China). The lyophilized samples were then ground into a fine powder using a Retsch MM400 grinding mill, operating at 30 Hz for 1.5 min. A precise aliquot of 50 mg of the resulting powder was measured on an MS105DU precision balance, placed into a tube, and combined with 1200 µL of a pre-cooled (−20 °C) extraction solution consisting of 70% methanol in water and an isotopically labeled internal standard. This mixture was subjected to six cycles of vortex mixing, each lasting 30 s, with 30 min intervals between cycles. Following centrifugation at 12,000 rpm for 3 min, the supernatant was retrieved, passed through a 0.22 µm membrane filter, and finally placed into an HPLC vial ready for UPLC-MS/MS analysis.

#### 2.2.2. HPLC Conditions

Separation was achieved chromatographically using an Agilent SB-C18 column (1.8 µm, 2.1 mm × 100 mm). The mobile phase consisted of (A) ultrapure water with 0.1% formic acid and (B) acetonitrile with 0.1% formic acid. The elution gradient was programmed as follows: starting at 5% B, ramping linearly to 95% B by 9.00 min, maintaining this level for 1 min, then returning to 5% B at 11.10 min, and allowing re-equilibration at 5% B until the 14.00 min mark. The method employed a flow rate of 0.35 mL/min, a column temperature of 40 °C, and an injection volume of 2 µL.

#### 2.2.3. Mass Spectrometry Conditions

For mass spectrometric detection, the ESI source was set to 500 °C, and the Ion Spray Voltage was applied at ±5500 V in both positive and negative modes. The pressures for Gas I, Gas II, and Curtain Gas were set at 50, 60, and 25 psi, respectively. Collision-induced dissociation was set to high. Analysis used a QQQ mass spectrometer operating in Multiple Reaction Monitoring (MRM) mode with nitrogen as collision gas at medium pressure. Declustering potentials and collision energies were optimized for each MRM transition. Specific MRM ion pairs were monitored according to metabolite retention times.

#### 2.2.4. Qualitative and Quantitative Analysis of Metabolites

Metabolite identification was conducted using a local metabolite database. In multiple reaction monitoring (MRM) mode, chromatograms displayed detectable compounds where each distinct peak represented a specific metabolite. Characteristic ions were identified by the triple-quadrupole mass spectrometer, and signal intensities were quantified as counts per second (CPS). The raw data were then processed with MultiQuant software by Applied Biosystems MDS Sciex (Foster City, CA, USA), version 3.0.3 to perform peak integration and necessary corrections. Here, peak areas corresponded to metabolite relative abundances.

### 2.3. Network Pharmacology

#### 2.3.1. Analysis of Potential Therapeutic Targets

The SMILES (Simplified Molecular Input Line Entry System) notations of the metabolites identified through *Ocimum* metabolomics were acquired from the PubChem database (https://pubchem.ncbi.nlm.nih.gov/; accessed on 18 June 2025). These SMILES were then submitted to the SwissTargetPrediction database (http://www.swisstargetprediction.ch/; accessed on 18 June 2025) to forecast potential protein targets in Homo sapiens, applying a probability score threshold of >0.1. The resulting metabolite-target relationships were used to build an interaction network, which was visualized with Cytoscape 3.9.1. Subsequently, the potential targets were subjected to protein–protein interaction (PPI) analysis on the STRING 12.0 platform (https://cn.string-db.org/; accessed on 19 June 2025), with the search confined to Homo sapiens and a high-confidence minimum interaction score of >0.9. To refine this network, the CytoHubba plugin within Cytoscape was employed to identify key candidate targets based on the Maximal Clique Centrality (MCC) algorithm. This refinement culminated in the construction of a core PPI network.

#### 2.3.2. Enrichment Analysis of Candidate Targets

The candidate targets derived from the above analysis underwent Gene Ontology (GO) enrichment analysis, covering biological processes, cellular components, and molecular functions as well as Kyoto Encyclopedia of Genes and Genomes (KEGG) pathway enrichment analysis. These analyses were conducted using the STRING 12.0 database (https://cn.string-db.org/; accessed on 20 June 2025). Visualization was subsequently performed using R version 4.2.0 following standard analytical workflows.

#### 2.3.3. Analysis of Medicinal Value of Metabolites

To evaluate the therapeutic potential, known disease associations for the candidate metabolites were acquired from the PubChem database. In parallel, targets linked to these diseases were sourced from the GeneCards database, applying a relevance score cutoff greater than 5 to ensure high-quality data. The final step involved integrating these data to build a comprehensive interaction network, which illustrated the intricate relationships between metabolites, targets, pathways, and diseases, using the visualization capabilities of Cytoscape 3.9.1 software.

### 2.4. Molecular Docking

The three-dimensional (3D) structures of the core target proteins were downloaded from the UniProt database (https://www.uniprot.org, accessed on 21 June 2025). Concurrently, the 3D conformational files of the candidate metabolites were obtained from PubChem. Molecular docking simulations were then conducted to predict binding modes, utilizing the CB-Dock2 web server (https://cadd.labshare.cn/cb-dock2/, accessed on 21 June 2025). The resulting docking poses, detailing the interactions between receptors (proteins) and ligands (metabolites), were visualized and analyzed using Discovery Studio 2019.

### 2.5. Evaluation of Antioxidant Efficacy

#### 2.5.1. Extract Preparation

Fresh basil leaves were pulverized in liquid nitrogen, and 0.2 g of the resulting powder was extracted with 4 mL of 60% ethanol using ultrasound-assisted extraction (40 kHz, 1 h). The extraction temperature was maintained below 40 °C. Subsequently, the mixture was centrifuged at 4000× *g* for 15 min. Finally, the supernatant was collected for antioxidant activity assessment.

#### 2.5.2. Antioxidant Activity Assays

All three antioxidant assays employed Trolox standard curves for quantification.

DPPH assay: 0.2 mL test solution was mixed with 2.8 mL of 0.1 mmol/L methanolic DPPH solution. After 30 min incubation in darkness at 25 ± 1 °C, absorbance was measured at 517 nm.

FRAP assay: The ferric reducing antioxidant power was determined by mixing 0.1 mL test solution with 4.9 mL freshly prepared FRAP reagent [containing 0.1 mol/L acetate buffer (pH 3.6); 10 mmol/L TPTZ (2,4,6-tripyridyl-s-triazine); and 20 mmol/L FeCl_3_ in 10:1:1 *v*/*v*/*v*]. Following 10 min dark incubation, absorbance was recorded at 593 nm.

ABTS assay: ABTS^+^ solution was generated by reacting 7 mmol/L ABTS with 140 mmol/L potassium persulfate for 12–16 h in darkness. Subsequently, 0.1 mL test solution was added to 3.9 mL ABTS^+^ solution. After 10 min dark incubation, absorbance was measured at 734 nm.

### 2.6. Data Statistics

Data were analyzed in Office 2021 (Microsoft Corporation, Redmond, WA, USA). while histogram generation was performed with Origin 2021 (OriginLab Corporation, Northampton, MA, USA). Visualization analyses, including Principal Component Analysis (PCA), classification pie charts, and petal Venn diagrams, were executed in R version 3.5.1. Additionally, K-means clustering analysis and heatmaps were produced utilizing R version 4.2.0.

The statistical framework incorporated both univariate and multivariate methods. Univariate analyses involved hypothesis testing and fold change (FC) calculation. Multivariate analyses primarily consisted of techniques such as PCA and orthogonal partial least squares-discriminant analysis (OPLS-DA). Metabolites exhibiting differential accumulation between accessions were initially screened based on variable importance in projection (VIP) values from the OPLS-DA model. To enhance robustness, this VIP-based screening was subsequently integrated with univariate criteria-specifically, the *p*-value/false discovery rate (FDR) (requiring ≥2 biological replicates) and the FC value-for the final identification of reliable differential metabolites.

## 3. Results

### 3.1. Overview of Identified Metabolites

**Figure 1:**
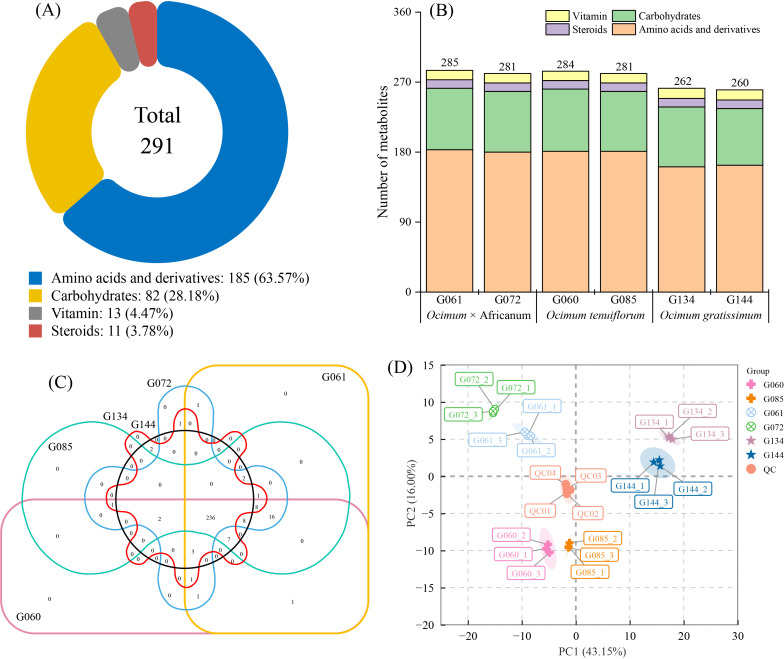
Overview of metabolites identified across different basil accessions. (**A**) Classification of the identified metabolites. (**B**) Number of metabolites identified per accession. (**C**) Venn diagram of metabolites identified per accession. (**D**) Principal component analysis (PCA) based on the identified metabolites.

Comprehensive metabolic profiling of six basil accessions across three species (*Ocimum* × africanum, *Ocimum tenuiflorum*, and *Ocimum gratissimum*) identified 291 nutritional metabolites, classified predominantly into amino acids and derivatives (185 metabolites, 63.60%), carbohydrates (82, 28.02%), vitamins (13), and steroids (11) (Figure 1A; Supplementary File S1: Table S1). Quantification revealed interspecific variation: *O.* × africanum (G061: 285; G072: 281) and *O. tenuiflorum* accessions (G060: 284; G085: 281) exhibited higher metabolite abundance than *O. gratissimum* (G134/G144: ~260 each) (Figure 1B). Venn analysis identified 236 universally shared metabolites and species-specific signatures: *O.* × africanum uniquely contained N-Methylisoleucine, while *O. gratissimum* possessed two exclusives (Cys-Pro-His and N-(beta-D-Glucosyl)nicotinate), no unique metabolites were detected in *O. tenuiflorum* or individual accessions (Figure 1C). Principal component analysis (PCA) explained 59.15% of total variance (PC1: 43.15%; PC2: 16.00%), demonstrating high reproducibility (tight clustering of QC/technical replicates) and significant interspecific divergence, The accessions clustered by species, with intraspecific pairs (*O.* × africanum: G061 and G072; *O. tenuiflorum*: G060 and G085; *O. gratissimum*: G134 and G144) showing closer proximity than interspecific groups, indicating greater metabolic conservation within species (Figure 1D).

### 3.2. Analysis of Differential Metabolites

**Figure 2:**
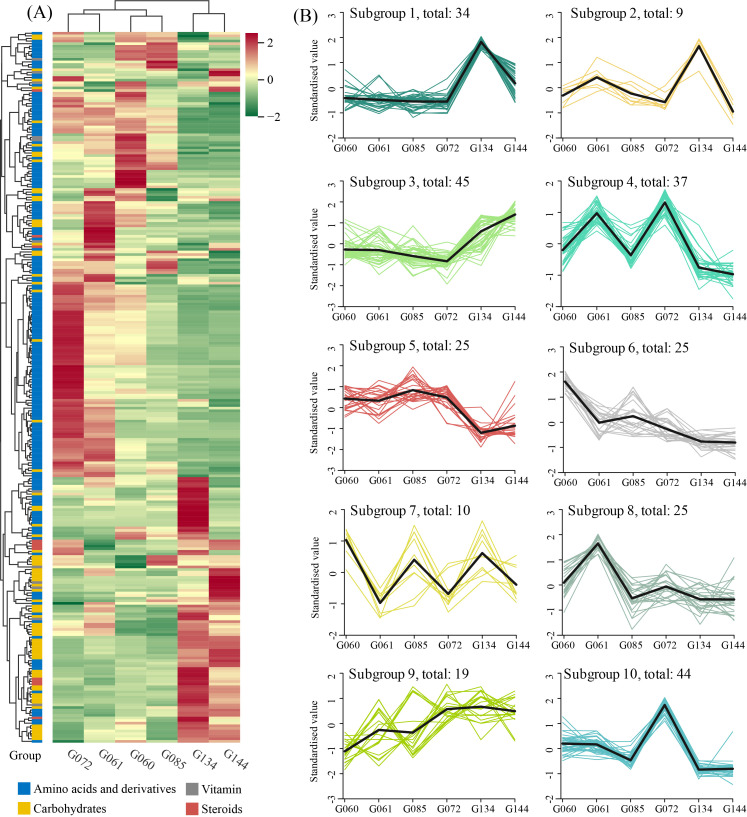
Analysis of differential metabolites among the materials. (**A**) Heatmap of the 273 identified DAMs. The color scale (from red to green) indicates the relative abundance (Z-score) of each metabolite, revealing distinct accumulation patterns that cluster the accessions by species. (**B**) K-means clustering analysis of the 273 DAMs, partitioned into 10 subgroups (1–10). Each line represents the standardized accumulation pattern of metabolites within a subgroup across the six accessions.

Applying stringent criteria, we identified 273 metabolites exhibiting significant differential accumulation (DAMs) across the six basil accessions. Hierarchical clustering analysis revealed distinct accumulation patterns for these DAMs among the accessions (Figure 2A). To elucidate co-regulated metabolite groups, K-means clustering partitioned the 273 DAMs into 10 distinct subgroups (1–10), ranging in size from 9 to 45 metabolites (Figure 2B). Subgroup 3 (n = 45, largest subgroup) displayed markedly high accumulation specifically within the *O. gratissimum* accessions (G134 and G144), with low levels observed in all other accessions. This suggests a species-specific metabolic signature for *O. gratissimum*. Subgroup 10 (n = 44) was characterized by pronounced accumulation uniquely in accession G072, indicating accession-specific metabolic traits. Subgroup 2 (n = 9, smallest subgroup) exhibited high accumulation exclusively in accession G134, pointing to potentially unique metabolic regulation within this specific *O. gratissimum* species.

**Figure 3:**
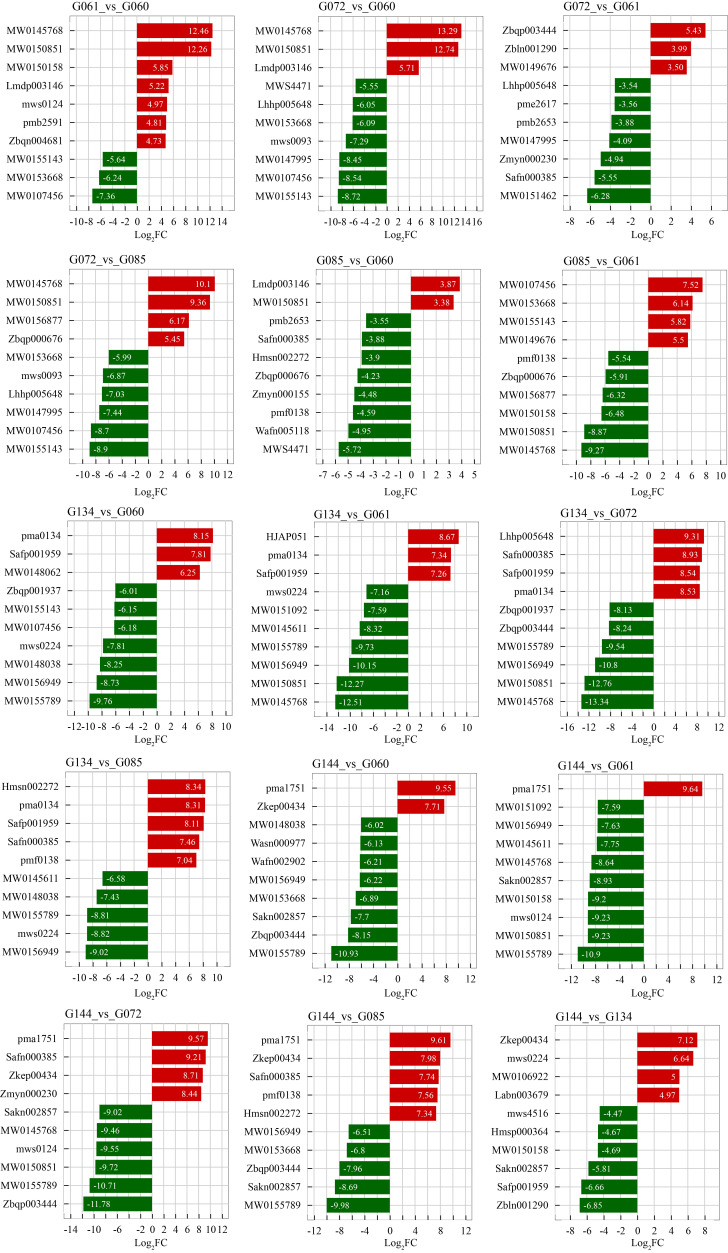
Top 10 significantly differential metabolites in each comparison group. For each of the 15 possible pairwise comparisons among the six accessions, the top 10 differential accumulated metabolites (DAMs) ranked by the absolute value of log_2_(fold change) are displayed. Metabolite names are shown on the y-axis, and the log_2_(FC) value is on the x-axis.

To systematically investigate the metabolic differences driving accession divergence, we performed pairwise comparisons across all 15 possible combinations of the six accessions. Each comparison group contained a variable number of DAMs. Venn diagram analysis identified a core set of metabolites consistently altered across the entire panel (Supplementary File S2: Figure S1): A single metabolite, Cys-Gln-Ala, was identified as differentially accumulated in all 15 pairwise comparisons. This highlights Cys-Gln-Ala as a highly conserved metabolic marker distinguishing these basil accessions under the studied conditions. Furthermore, 11 out of the 15 pairwise groups contained unique DAMs not shared with any other comparison (Supplementary File S2: Figure S1), underscoring the presence of highly specific metabolic differences between particular accession pairs. To prioritize metabolites exhibiting the most substantial changes for further investigation, we visualized the top 10 DAMs ranked by the magnitude of fold change (|log_2_(FC)|) for each of the 15 pairwise comparisons (Figure 3). This analysis revealed several metabolites consistently appearing among the top differential accumulators in multiple comparisons: key examples include the tripeptide Asn-His-Gly and N-(beta-D-Glucosyl)nicotinate, suggesting their potential significance in defining broader metabolic distinctions within the basil panel. Compiling these top-ranking metabolites from all pairwise comparisons resulted in an initial list of 150 metabolites (15 comparisons × 10 DAMs). Subsequent removal of duplicates and combining the common and unique DAMs yielded 61 unique high-impact candidate DAMs. These candidate metabolites represent the most dynamically altered compounds across the studied accessions and were selected for subsequent functional and pathway analyses.

### 3.3. Target Prediction and PPI Network Refinement

**Figure 4:**
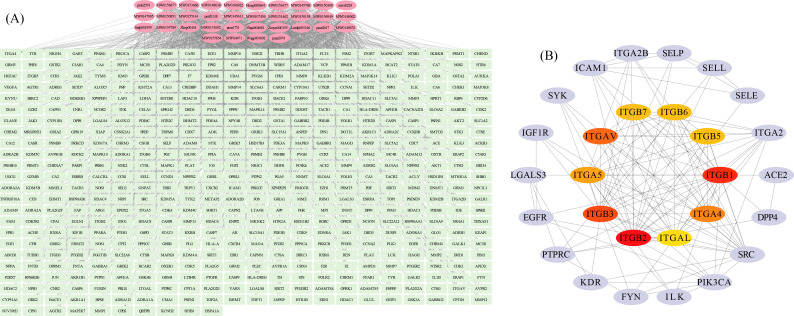
Prediction of potential targets for candidate differential metabolites. (**A**) Regulatory network diagram of candidate differential metabolites and their potential targets. Green and pink nodes denote targets and metabolites, respectively. (**B**) Protein–protein interaction (PPI) network analysis of putative targets. Purple nodes represent all targets within the PPI network, while orange-red nodes highlight the top 10 hub targets identified.

Based on metabolomic profiling, a total of 61 candidate DAMs were initially identified. Subsequent target prediction analysis revealed that 47 of these metabolites possessed potential biological targets (Figure 4A). Candidate functional metabolites included 4-O-galactopyranosylxylose, D-Threose, Gln-Glu-Asp, Coniferyl alcohol, L-Methionine Sulfoxide, and multiple di-/tri-peptides (e.g., Lys-Asp, Asp-Lys, Met-Thr-His), along with specialized metabolites such as Intergristerone A-22-O-galactoside (Sileneoside C) and Jasmonoyl-L-Isoleucine. These metabolites collectively predicted 516 potential targets, encompassing a broad spectrum of proteins involved in signaling, metabolism, and cellular regulation. Notable targets spanned receptors (e.g., NTSR1, OPRM1, CXCR4), enzymes (e.g., ACE, PTGS2, HMGCR), kinases (e.g., AKT1, SRC, MAPK14), epigenetic regulators (e.g., EZH2, KDM4A), and immune-related molecules (e.g., HLA-A, HLA-DRB1), highlighting their diverse functional roles in biological pathways.

Protein–protein interaction (PPI) network analysis of the 516 predicted targets refined the candidate pool to 28 high-confidence targets (Figure 4B). These core targets including IGF1R, KDR, EGFR, PIK3CA, SRC, FYN, DPP4, ACE2, and integrin family members (ITGA2, ITGA5, ITGAV, ITGB1-7) are critically associated with cell adhesion, growth signaling, and inflammatory responses. The prominence of integrins (e.g., ITGA2B, ITGB3) and immune regulators (e.g., ICAM1, SELP, SELE, PTPRC) suggests a central role in extracellular matrix dynamics and leukocyte trafficking. Additionally, kinases (e.g., SYK, ILK) and receptors (e.g., IGF1R, KDR) underscore pathways driving proliferation and angiogenesis. This refined target set provides a focused framework for elucidating mechanistic links between metabolic perturbations and disease-relevant cellular processes.

### 3.4. Enrichment Analysis

**Figure 5:**
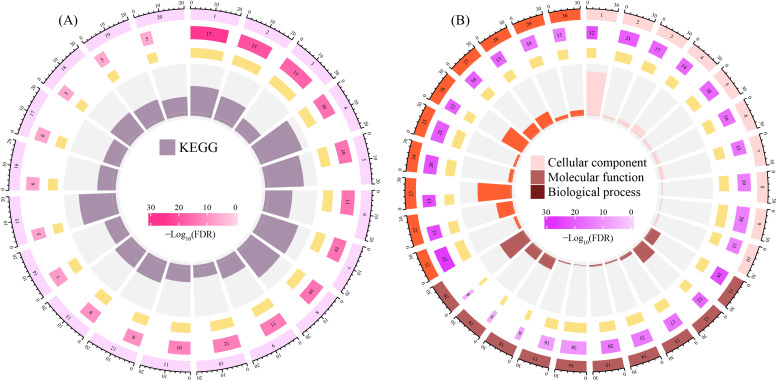
Functional enrichment analysis of the 28 core target proteins. (**A**) Kyoto Encyclopedia of Genes and Genomes (KEGG) pathway enrichment analysis. The most significantly enriched pathways are shown, with the rich factor (**B**) Gene Ontology (GO) enrichment analysis across Biological Process (BP), Cellular Component (CC), and Molecular Function (MF) categories. The top significantly enriched terms are displayed.

KEGG enrichment analysis of the 28 core targets revealed significant associations with focal adhesion, ECM–receptor interaction, PI3K–Akt signaling, and pathways related to cardiomyopathy, platelet activation, and immune trafficking (Figure 5A). These findings suggest that basil primary metabolites may influence integrin-mediated processes relevant to cardiovascular protection, anti-metastatic signaling, and immunomodulation.

GO enrichment analysis further supported these observations, with strong enrichment in cellular components such as integrin complex and focal adhesion, molecular functions including integrin binding and extracellular matrix binding, and biological processes like cell migration and integrin-mediated signaling (Figure 5B). Together, these results highlight the potential of basil metabolites to modulate cell-adhesion dynamics, extracellular matrix integrity, and immune–vascular crosstalk.

### 3.5. Core Metabolite Therapeutic Value Analysis

**Figure 6:**
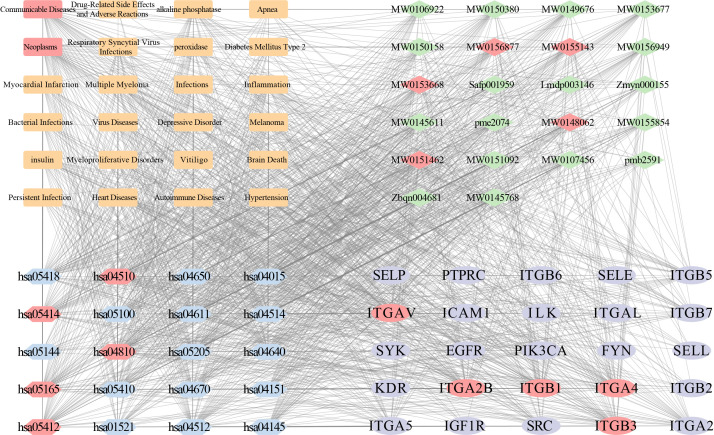
Integrated regulatory network diagram illustrating candidate differential metabolites, their potential targets, enriched pathways, and associated diseases. In the network, orange, green, blue, and purple nodes denote diseases, metabolites, pathways, and targets, respectively. Red nodes represent hubs selected according to the Maximal Clique Centrality (MCC) algorithm.

Integrative network analysis connected 22 candidate metabolites to 24 diseases, 25 core targets (mainly integrins and kinases), and 20 enriched pathways centered on cell adhesion, signaling dysregulation, and immune–pathogen interactions (Figure 6). This network positions the integrin–cytoskeleton–immune axis as a key therapeutic interface.

Five hub metabolites, including Ser-Phe-Met, Cys-Pro-His, Phe-Cys-Gln, Ile-His-Val, and Met-Ser-Tyr were identified as multi-pathway regulators of integrin signaling. Their structural features (e.g., cysteine-rich or metal-coordinating motifs) may facilitate binding to integrin subunits (ITGB1, ITGB3, ITGA4, ITGA2B, ITGAV), potentially influencing anti-metastatic, anti-infective, and cardioprotective processes. These metabolites represent promising scaffolds for further development of integrin-targeted therapeutics candidates.

### 3.6. Molecular Docking

Based on prior functional enrichment analyses implicating integrin-mediated signaling pathways, we performed molecular docking simulations between five key differential tripeptides (Ile-His-Val, Cys-Pro-His, Phe-Cys-Gln, Met-Ser-Tyr, Ser-Phe-Met) and five integrin subunits (ITGB1, ITGB3, ITGA4, ITGA2B, ITGAV) identified as hub targets. These integrins were prioritized due to their established roles in cell adhesion, migration, and inflammatory responses processes potentially modulated by bioactive peptides. Docking simulations were conducted. All docked complexes exhibited negative binding energies (<−5 kcal/mol) (Supplementary File S3: Table S2), indicating potential spontaneous and thermodynamically favorable binding. Notably, several complexes demonstrated exceptionally strong binding (<−7 kcal/mol), suggesting possible high-affinity interactions that may be relevant to biological activity: ITGB1 Complexes: Met-Ser-Tyr displayed the strongest affinity (binding energy = −7.4 kcal/mol), significantly outperforming other peptides. This suggests a potentially privileged interaction with ITGB1, a subunit critically involved in angiogenesis and cancer progression. ITGB3 Complexes: Met-Ser-Tyr also showed the highest stability with ITGB3 (binding energy = −7.0 kcal/mol), a key platelet receptor and therapeutic target in thrombosis. ITGA4/ITGA2B/ITGAV Complexes: Phe-Cys-Gln emerged as the dominant binder for these subunits: ITGA4: binding energy = −7.9 kcal/mol (implicated in lymphocyte homing and autoimmune diseases). ITGA2B: binding energy = −8.1 kcal/mol (critical for platelet aggregation). ITGAV: binding energy = −8.5 kcal/mol (associated with tumor growth and metastasis).

**Figure 7:**
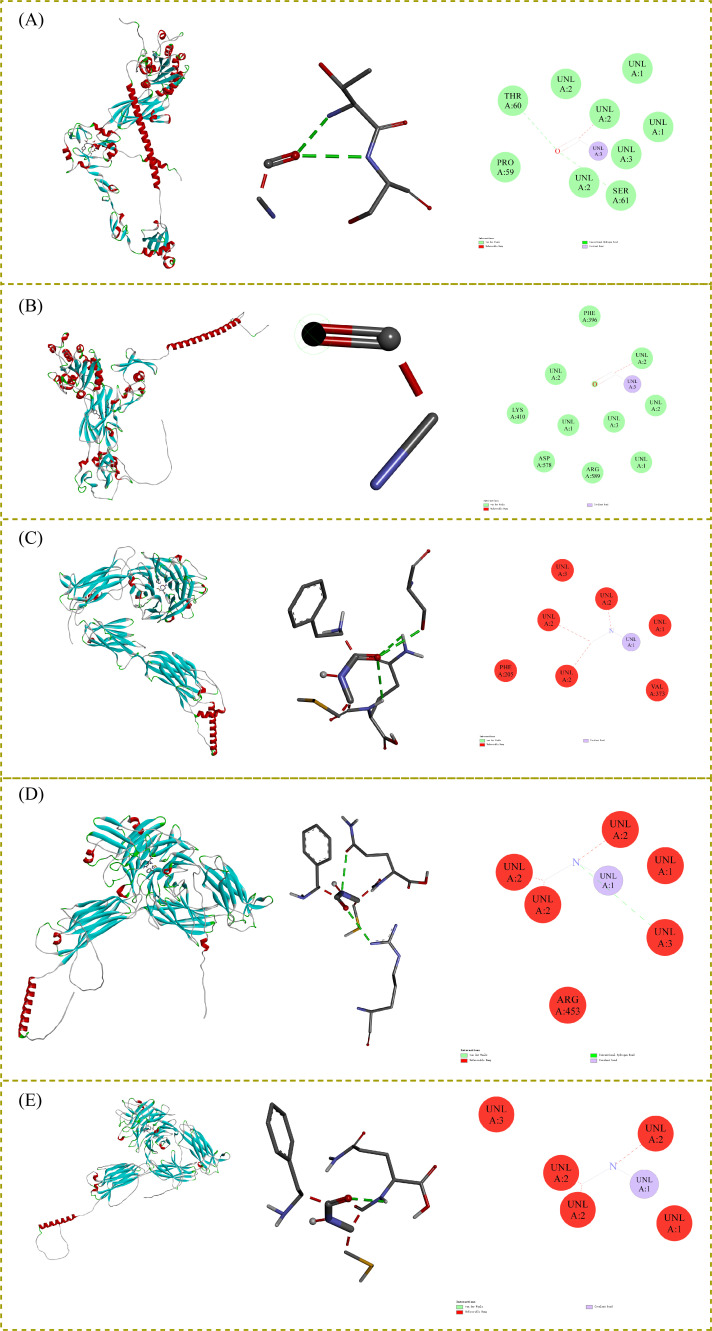
Molecular docking conformation of functional nutritional metabolites with key targets in *Ocimum.* (**A**) Met-Ser-Tyr with ITGB1. (**B**) Met-Ser-Tyr with ITGB3. (**C**) Phe-Cys-Gln with ITGA4. (**D**) Phe-Cys-Gln with ITGA2B. (**E**) Phe-Cys-Gln with ITGAV.

Detailed analysis of binding modes revealed the molecular basis for the observed affinities (Figure 7): Met-Ser-Tyr with ITGB1 (Figure 7A) and ITGB3 (Figure 7B): Stabilization primarily involves van der Waals forces, conventional hydrogen bonds (e.g., with key residues Asp, Arg in the metal ion-dependent adhesion site-MIDAS), and covalent interactions (potentially coordinating divalent cations like Mg^2+^ essential for integrin function). Minor unfavorable steric clashes were observed but outweighed by stabilizing forces. The strong interaction with ITGB1/ITGB3 suggests potential modulation of pathways related to cell adhesion, migration, or platelet activation. Phe-Cys-Gln with ITGA4 (Figure 7C), ITGA2B (Figure 7D), and ITGAV (Figure 7E): Binding is driven by extensive van der Waals contacts, covalent interactions (potentially disulfide bridging or cation coordination via cysteine and glutamine side chains), and localized unfavorable bumps. The remarkably low energy with ITGAV (binding energy = −8.5 kcal/mol) warrants particular attention, as αV integrins are validated therapeutic targets in fibrosis and oncology. The presence of cysteine suggests potential redox sensitivity in its binding mechanism.

### 3.7. Evaluation of Antioxidant Efficacy

**Figure 8:**
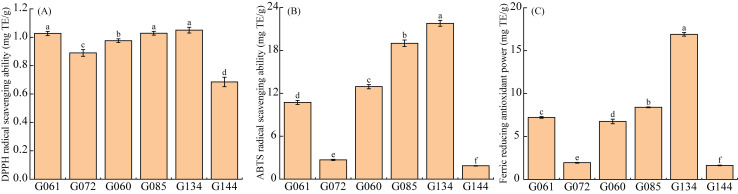
Analysis of antioxidant capacity across different Basil accessions. (**A**) DPPH radical scavenging ability. (**B**) ABTS radical scavenging ability. (**C**) Ferric reducing antioxidant power. Data are expressed as the mean ± standard error (SE) of three biological replicates (n = 3). Different lowercase letters above the bars denote statistically significant differences among accessions, as determined by one-way ANOVA followed by Duncan’s multiple range test (*p* < 0.05). Specifically, accessions labeled with the same letter are not significantly different from each other, whereas those labeled with different letters are statistically different.

The antioxidant efficacy of six distinct basil accessions was comprehensively evaluated using three established assays: DPPH radical scavenging, ABTS radical scavenging, and ferric reducing antioxidant power (FRAP) (Figure 8). While all accessions exhibited measurable antioxidant activity across assays, significant inter-genotypic variations were observed. Specifically, DPPH radical scavenging capacity (Figure 8A) represented the weakest response among the three metrics, with limited variability; accessions G134, G085, and G061 formed a statistically homogeneous high-performance subgroup, yet significantly outperformed (*p* < 0.05) the remaining three accessions. In stark contrast, ABTS radical scavenging capacity was exceptionally robust across all accessions (Figure 8B), with all six accessions showing significant pairwise differences (*p* < 0.05). Similarly, FRAP assays revealed strong electron-donating activity comparable to ABTS results (Figure 8C), though exhibiting distinct genotypic ranking patterns while maintaining significant inter-genotypic differences (*p* < 0.05). Collectively, the markedly superior performance in ABTS and FRAP assays, attributable to basil’s abundance of hydrophilic antioxidants highlights their utility for discriminating antioxidant-rich genotypes, whereas the attenuated DPPH response suggests limited lipophilic radical neutralization. Notably, the consistent efficacy of G134, G085, and G061 positions them as prime candidates for further phytochemical exploration.

## 4. Discussion

Basil, as an important medicinal and edible plant, warrants significant research attention regarding its primary metabolites [38]. In this study, UPLC-MS/MS analysis was employed to quantify amino acids, vitamins, carbohydrates, and steroidal metabolites across six distinct basil accessions, yielding comprehensive datasets. Regarding amino acids, numerous common types, including both essential and non-essential amino acids, were detected. Substantial variations in amino acid profiles were observed among the accessions. Notably, several demonstrated advantages in providing essential amino acids for human nutrition; furthermore, these differences highlight their divergent potential for medicinal exploitation based on amino acid composition [39,40]. Similarly, diverse vitamins and common sugars were identified during the analysis of vitamins and carbohydrates. Moreover, the composition and concentration of these metabolites varied considerably across materials, potentially attributable to their specific varietal characteristics. The pronounced disparities in carbohydrates not only influence their energy-supplying properties but also likely affect organoleptic qualities [39,40]. Significant differences in both the types and levels of steroidal metabolites were also evident among the basil accessions. These variations are likely intrinsically linked to the plant’s medicinal value and physiological functions [41]. Steroidal metabolites play crucial roles in regulating plant growth and development, enhancing resistance to pests and diseases, and conferring medicinal benefits to humans [42]. Consequently, the observed variations in their content may lead to significant differences in the medicinal and health-promoting efficacy of different basil materials.

Beyond basil, studies on plant-derived amino acids, vitamins, carbohydrates, and steroidal metabolites in species such as *Camellia fascicularis*, *Lycium barbarum*, *Cinnamomum* spp., *Polygonatum cyrtonema*, and *Brassica oleracea* have illustrated the therapeutic potential of these compounds, emphasizing their roles in antioxidant, antimicrobial, metabolic regulation, and immunomodulatory activities [43,44,45,46,47]. Specifically: Amino Acids/Peptides: Bioactive peptides (e.g., antioxidant peptides) can modulate physiological functions such as blood pressure regulation, immune response, and antimicrobial defense. They also serve as precursors to signaling molecules and exhibit metal-chelating properties [48]. Vitamins: Vitamin C (ascorbic acid) and B-group vitamins (e.g., B1, B6) contribute to plant stress resistance and human health through antioxidant activity, acting as enzyme cofactors, and supporting immune function. Vitamin K facilitates calcium deposition in bones [49]. Carbohydrates: Polysaccharides (e.g., arabinogalactans, fructans) may improve gut microbiota composition, stimulate the production of short-chain fatty acids (SCFAs), and enhance immune function via prebiotic effects. They are often resistant to gastrointestinal digestion and can exert systemic anti-inflammatory effects [50]. Steroidal Metabolites: C21 steroidal compounds and phytosterols (e.g., β-sitosterol) demonstrate anticancer, cholesterol-lowering, and anti-inflammatory activities primarily by modulating enzymatic pathways and hormone receptors, supporting their therapeutic applications [51].

Molecular docking reveals a notable prevalence of covalent interactions among the top-ranked complexes. While covalent binding can confer high specificity and prolonged residence time, a desirable trait in drug design, it however necessitates careful evaluation of selectivity and potential off-target effects in future studies [52]. The consistent involvement of residues critical for integrin activation (e.g., within the MIDAS domain) further supports the potential functional relevance of these interactions [53]. Regarding Met-Ser-Tyr: Its strong, selective binding to β1 and β3 integrins positions it as a candidate modulator of angiogenesis (via ITGB1), thrombosis (via ITGB3/ITGA2B), or osteoclast function (via ITGAV/ITGB3). The Tyr residue may facilitate interactions that mimic RGD-like motifs [54]. Regarding Phe-Cys-Gln: Its broad, high-affinity binding to multiple α-subunits (ITGA4, ITGA2B, ITGAV) suggests potential as a pan-integrin modulator. The Cys-Gln sequence is structurally intriguing, potentially mimicking aspects of disintegrin domains or coordinating integrin metal ions. Its strong affinity for ITGAV additionally highlights potential relevance in cancer metastasis or fibrosis [55]. Collectively, these computationally predicted high-affinity interactions between specific basil-derived tripeptides and integrin subunits provide a compelling mechanistic hypothesis for their potential pharmacological effects, particularly in inflammation, thrombosis, and cell adhesion processes [56].

Met-Ser-Tyr, composed of methionine (Met), serine (Ser), and tyrosine (Tyr), exhibits neuroregulatory and metabolic functions, where Tyr serves as a neurotransmitter precursor (e.g., for dopamine and norepinephrine), Met acts as a methyl donor for methylation reactions, and Ser participates in phospholipid synthesis and signal transduction [57]; pharmacologically, it demonstrates neuroregulatory potential by potentially improving cognitive function through Tyr-derived neurotransmitters, analogous to the effects of Val/Met variants in catechol-O-methyltransferase (COMT) gene polymorphism studies on cognition, alongside antioxidant and metabolic modulation wherein Met’s sulfur atom neutralizes reactive oxygen species (ROS) and Ser contributes to gluconeogenesis, synergistically regulating energy metabolism [58]. Conversely, Phe-Cys-Gln containing phenylalanine (Phe), cysteine (Cys), and glutamine (Gln) with Cys enabling disulfide bond formation via its sulfhydryl group (-SH) and Gln providing nitrogen for immune modulation shows defense-eliciting and immunoenhancing properties, functioning as an elicitor akin to the non-proteinogenic amino acid β-Tyr (3-amino-3-(4-hydroxyphenyl)propionic acid) to activate the jasmonic acid (JA) biosynthesis pathway and enhance insect resistance, while its Cys promotes glutathione synthesis to bolster oxidative stress tolerance and Gln supports intestinal barrier integrity, suggesting anti-inflammatory reparative potential [59,60,61]. Collectively, Met-Ser-Tyr and Phe-Cys-Gln confer core potential pharmacological value by modulating neurotransmitter synthesis, activating JA defense pathways, and enhancing antioxidant capacity, with efficacy validated in crops like rice [62] and tomato [63] involving crosstalk with conserved CPK28-MAPK or JA signaling cascades, necessitating future research to elucidate receptor-binding mechanisms and develop delivery systems for advancing agricultural and pharmaceutical applications [64].

While providing initial insights into primary metabolite diversity across six *Ocimum* accessions and predicting bioactivity mechanisms (e.g., integrin modulation), this study has limitations: its restricted species/accession scope necessitates broader germplasm analysis; predicted bioactivities require in vitro (e.g., adhesion/migration assays) and in vivo disease model validation; metabolite stability and bioavailability remain unassessed; and potential synergistic effects between metabolites or precise functional consequences of interactions (e.g., peptide-integrin binding) are unexplored. Consequently, future research should: validate predicted bioactivities (e.g., anti-adhesion, immunomodulation) of key metabolites (e.g., Met-Ser-Tyr, Phe-Cys-Gln) in relevant models (e.g., cancer, inflammation); investigate ADME profiles of bioactive metabolites (especially tripeptides); leverage pathway knowledge for metabolic engineering or elite cultivar development; assess efficacy/safety of enriched extracts or isolated compounds (notably peptides) in human trials, developing optimized delivery systems; and explore synergy among primary metabolites and between primary/secondary metabolites. Finally, beyond mechanistic validation, this study opens several translational avenues for advancing basil as a medicinal plant. Future efforts should include the development of standardized basil cultivars or extracts with optimized primary metabolite profiles through targeted breeding or cultivation strategies. Integrating these findings with agronomic practices could enhance the consistent production of bioactive primary metabolites. Furthermore, establishing quality control markers from primary metabolite signatures would support the reliable use of basil in nutraceuticals and herbal formulations. Exploring the interplay between primary and secondary metabolites in whole-plant extracts may also reveal synergistic effects that enhance overall bioactivity, bridging traditional use with modern evidence-based applications.

## 5. Conclusions

This study provides a novel perspective on basil (*Ocimum* spp.) by demonstrating that its primary metabolites, traditionally viewed only as nutritional constituents possess significant functional and therapeutic potential. By integrating metabolomics, antioxidant evaluation, and network-based molecular analyses, we show that amino acids, small peptides, vitamins, and carbohydrates contribute to bioactivity through integrin-related pathways and antioxidant mechanisms. These results challenge the conventional focus on secondary metabolites and highlight primary metabolites as potential active modulators of processes relevant to immunity, inflammation, and disease prevention. Based on our comparative profiling, preliminary functional insights can be inferred for the studied species: *Ocimum* × africanum and *O. tenuiflorum* accessions, with their higher overall primary metabolite abundance and strong antioxidant performance (e.g., G085, G061), may be prioritized for developing broad-spectrum antioxidant and nutritive supplements. In contrast, *O. gratissimum* accessions (G134, G144) displayed unique metabolite signatures (e.g., specific tripeptides) and a distinct antioxidant profile, suggesting a potentially different application niche, possibly aligned with the integrin-targeting activities predicted for their specific metabolites. However, we emphasize that these are preliminary, metabolome-based inferences. The novelty of this work lies in establishing primary metabolites as a fundamental and underexplored layer of basil’s bioactivity. It provides a hypothetical mechanistic basis for future, more targeted investigations. Crucially, a definitive practical application strategy for specific basil species will require an integrated analysis combining both primary and secondary metabolome data, alongside validation in biological models. Moving forward, such multi-layered profiling, validation in cellular and animal models, and targeted breeding of metabolite-rich cultivars will be essential for translating these insights into tailored, evidence-based health applications.

## Data Availability

The data will be made available from the corresponding author upon reasonable request.

## Supplementary Materials

The following supporting information can be downloaded at: https://www.mdpi.com/article/10.3390/life16020273/s1, Supplementary File S1: Table S1. Parameters for the identification of primary metabolites in three *Ocimum* species using UHPLC-ESI-QTRAP-MS/MS. Supplementary File S2: Figure S1. Petal venn diagram. Supplementary File S3: Table S2. Binding energy (kcal/mol).
