# Supplementary figures and images for: Primary Metabolites in Three Ocimum Species: Compositional Diversity, Network Pharmacology, and Integrin-Targeted Therapeutic Implications
Source: Life (Basel). 2026 Feb 4;16(2):273. doi: 10.3390/life16020273 (PMC12942066; doi:10.3390/life16020273)

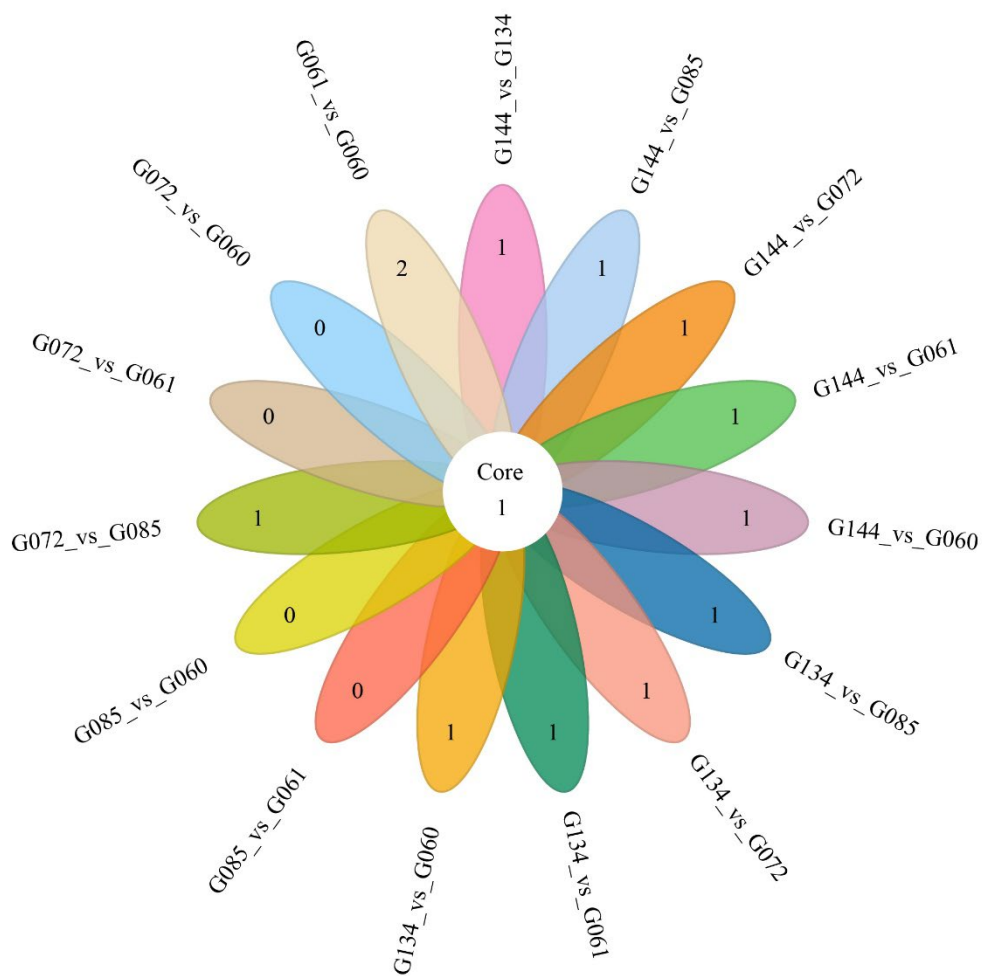

Supplementary File S2: Figure S1. Petal venn diagram.

Supplement: Supplementary file 1 [file life-16-00273-s001.zip › Supplementary File S2 Figure S1.pdf]
